# Increased CD39 Nucleotidase Activity on Microparticles from Patients with Idiopathic Pulmonary Arterial Hypertension

**DOI:** 10.1371/journal.pone.0040829

**Published:** 2012-07-11

**Authors:** Scott H. Visovatti, Matthew C. Hyman, Diane Bouis, Richard Neubig, Vallerie V. McLaughlin, David J. Pinsky

**Affiliations:** 1 Division of Cardiology, Department of Medicine, University of Michigan, Ann Arbor, Michigan, United States of America; 2 Department of Medicine, University of Pennsylvania, Philadelphia, Pennsylvania, United States of America; 3 Department of Pharmacology, University of Michigan, Ann Arbor, Michigan, United States of America; University of Colorado Denver, United States of America

## Abstract

**Background:**

Idiopathic pulmonary arterial hypertension (IPAH) is a devastating disease characterized by increased pulmonary vascular resistance, smooth muscle and endothelial cell proliferation, perivascular inflammatory infiltrates, and *in situ* thrombosis. Circulating intravascular ATP, ADP, AMP and adenosine activate purinergic cell signaling pathways and appear to induce many of the same pathologic processes that underlie IPAH. Extracellular dephosphorylation of ATP to ADP and AMP occurs primarily *via* CD39 (ENTPD1), an ectonucleotidase found on the surface of leukocytes, platelets, and endothelial cells [1]. Microparticles are micron-sized phospholipid vesicles formed from the membranes of platelets and endothelial cells. *Objectives*: Studies here examine whether CD39 is an important microparticle surface nucleotidase, and whether patients with IPAH have altered microparticle-bound CD39 activity that may contribute to the pathophysiology of the disease.

**Methodology/ Principal Findings:**

Kinetic parameters, inhibitor blocking experiments, and immunogold labeling with electron microscopy support the role of CD39 as a major nucleotidase on the surface of microparticles. Comparison of microparticle surface CD39 expression and nucleotidase activity in 10 patients with advanced IPAH and 10 healthy controls using flow cytometry and thin layer chromatograph demonstrate the following: 1) circulating platelet (CD39^+^CD31^+^CD42b^+^) and endothelial (CD39^+^CD31^+^CD42b^−^) microparticle subpopulations in patients with IPAH show increased CD39 expression; 2) microparticle ATPase and ADPase activity in patients with IPAH is increased.

**Conclusions/ Significance:**

We demonstrate for the first time increased CD39 expression and function on circulating microparticles in patients with IPAH. Further research is needed to elucidate whether these findings identify an important trigger for the development of the disease, or reflect a physiologic response to IPAH.

## Introduction

Idiopathic pulmonary artery hypertension (IPAH) is a devastating disease of unclear etiology that ultimately results in right-sided heart failure, low cardiac output and death. Though the exact etiology of IPAH remains unclear, the disease is characterized by increased pulmonary vascular resistance, smooth muscle and endothelial cell proliferation, perivascular inflammatory infiltrates, and *in situ* thrombosis. The contributions of prostanoids, endothelin-1, nitric oxide, BMPR-II signaling, endothelial dysfunction, and inflammation to the pathobiology of IPAH have been studied extensively, and such investigations form the basis of current treatment options [2]. Despite such advances, median survival in the modern treatment era is 3.6 years [3], increased from a mean survival of 2.8 years in untreated patients [4]. Thus, continued investigation into the mechanisms leading to the development of IPAH is of paramount importance.

In addition to their roles as intracellular energy transporters, the purine nucleotides ATP, ADP, and AMP are important extracellular signaling molecules [5]. Such purinergic signaling is vital to the regulation of processes including vessel tone [6], apoptosis [7], smooth muscle and endothelial cell proliferation [8], platelet aggregation [9], and inflammation [10]; many of the same processes that are dysregulated in IPAH. Thus, it is possible that a perturbation in purinergic signaling due to a disruption in the normal intravascular nucleotide balance may play a role in the pathophysiology of IPAH.

Regulation of nucleotides within the extracellular, intravascular milieu is regulated in large part through the enzymatic activity of the transmembrane ectonucleotidase CD39 (ENTPD1) [11], [12]. Studies have shown that CD39-mediated dephosphorylation of ATP and ADP into AMP plays a central role in vital homeostatic processes including thromboregulation [13], inflammation, stroke and the immune response [14], and apoptosis [15]. CD39 localizes to the surface of endothelial cells, circulating platelets, and some leukocytes [1]. Recently, CD39 activity has also been reported on circulating microparticles [16]. Microparticles are micron-sized phospholipid vesicles shed from cell membranes in response to activation or apoptosis [17]. The majority of circulating microparticles are thought to derive from platelets, with endothelial cell-, erythrocyte-, and leukocyte-derived microparticles contributing smaller percentages to the total circulating pool [18]. Each microparticle carries on it surface proteins specific to the membranes of the parent cell. The composition and structure of microparticles make them a unique circulating repository of potentially bioactive molecules, and studies have supported the participation of microparticles in diverse biological functions including thrombosis [19], hematopoiesis [20], and inflammation [21].

**Table 1:** Sample Characteristics.

		Controls (N = 10)	IPAH (N = 10)
Age (in years, Mean, Range)		41 (28 – 55)	47 (15 – 63)
Gender (N)			
	Males	0	0
	Females	10	10
Current Tobacco Use (N)			
	Yes	0	0
	No	10	10
Platelet Count (Mean, SEM)		197±31×10^9^	183×10^9^±34×10^9^

**Table 2:** Clinical, Functional and Hemodynamic Characteristics of IPAH Patients (N = 10).

World Health Organization Class		
	Class IV	100%
IPAH-specific Therapy		
	Parenteral Prostacyclin Therapy	100%
	Phosphodiesterase-5 Inhibitor	43%
	Endothelin-1 receptor Blocker	57%
Anticoagulation Therapy		
	Aspirin	16%
	Warfarin	59%
Functional		
	6-minute walking distance	170±33 meters
Hemodynamic		
	Mean Right Atrial Pressure	13.0±2.9 mm Hg
	Mean Pulmonary Arterial Pressure	54.8±5.7 mm Hg
	Mean Pulmonary Capillary Wedge Pressure	12.1±1.0 mm Hg
	Cardiac Output*	4.4±0.9L/min
	Cardiac Index*	1.84±0.4 L/min/m^2^
	Pulmonary Vascular Resistance	11.8±1.7 Wood Units

* Thermodilution method used. All values are expressed as mean ± standard error of the mean.

We hypothesize that circulating platelet and endothelial microparticles in patients with IPAH exhibit altered surface CD39 expression and function. If this hypothesis is validated, it would implicate altered purinergic signaling in the pathogenesis of IPAH, or activation of associated compensatory mechanisms.

## Methods

### Reagents used

HEPES buffer, TWEEN-20, isobutanol, isoamyl alcohol, 2-ethoxyethanol, ammonia, β-glycerophosphate, ammonium molybdate, levamizole, ouabain, oligomycin, and formic acid were obtained from Sigma-Aldrich (St. Louis, MO). M-270 epoxy Dynabeads, phosphate buffered saline, and RPMI containing 2.05 mM L-glutamine were purchased from Invitrogen (Carlsbad, CA). Six nanometer gold particles and gluteraldehyde phosphate buffer were purchased from Electron Microscopy Sciences (Hatfield, PA). Antibodies were purchased from the following vendors: mouse anti-human CD42b, FITC-conjugated mouse anti-human CD42b, APC-conjugated mouse anti-human CD31 and appropriate isotype controls (eBioscience, San Diego, CA); PE-conjugated mouse anti-human CD39 antibody and isotype control (Ancell, Bayport, MN); anti-CD39 clone A1, anti-ENTPD2 clone EPR3885, polyclonal anti-ENTPD3 and polyclonal anti-ENTPD8 (Abcam, Cambridge, MA). The inhibitors suramin, ARL 67156, and POM-1 were purchased from R&D Systems (Minneapolis, MN). HistoGel was obtained from Thermo Scientific (Asheville, NC). For flow cytometry experiments CaliBRITE beads, FluoSphere fluorescent beads, Annexin V Binding Buffer, APC-conjugated annexin V, and TruCOUNT tubes were purchased from BD Biosciences (San Jose, CA). 14C-radiolabeled radiochemicals were purchased from the following vendors: ATP and ADP (MP Biomedicals, Solon, OH); ADP and adenosine (Perkin Elmer, Waltham, MA).

**Table 3:** Influence of inhibitors on relative ATPase and ADPase activity.

		ATPase		ADPase	
Inhibitor	Concentration	Control	IPAH	Control	IPAH
No inhibitor	-	100±3.2	100±1.2	100±2.4	100±6.8
β-glycerophosphate	10 mM	136±5.6	124±3.5	110±3.2	114±5.4
Ammonium molybdate	1 mM	101±4.7	92±6.2	98±6.7	94±4.9
Levamizole	1 mM	82±2.9	89±4.7	90±7.1	88±3.2
Ouabain	1 mM	117±5.1	104±2.7	102±4.8	92±8.8
Oligomycin	10 μg mL^−1^	142±6.1	104±4.4	132±8.4	107±7.7
Suramin	0.5 mM	20±.0.1*	12±0.1*	15±0.5*	9±1*
ARL 67156	100 μM	22±0.1*	9±02*	14±0.3*	4±0.2*
POM-1	100 μM	7±0.1*	6±0.1*	1±0.1*	7±0.2

Relative nucleotidase activities expressed as percentage of available substrate dephosphorylated, and is normalized to reactions without inhibitors. The experiment was performed in triplicate using isolated microparticles from three healthy controls and three IPAH patients. *Significant difference from the sample without inhibitor (*P*<0.05).

### Patients and control subjects

The study protocol was approved by the Institutional Review Boards (IRBs) of the University of Michigan, Baylor University, Stanford University and the Cleveland Clinic. All subjects provided written informed consent. Patients who had been diagnosed with IPAH, and who were active on lung or heart-lung transplant lists, were approached for participation in the project. Inclusion criteria for patient participants were as follows: 1) a diagnosis of IPAH; 2) mean pulmonary artery pressure (mPAP) >25 mm Hg; 3) pulmonary capillary wedge pressure or left ventricular end diastolic pressure ≤15 mmHg; 4) pulmonary vascular resistance >3.0 Wood units; 5) total lung capacity >60% predicted; 6) Doppler echocardiogram demonstrating no significant mitral valve, aortic valve, or left heart disease; 7) pulmonary function testing demonstrated the absence of significant obstructive lung disease. Ten patients with idiopathic pulmonary arterial hypertension were recruited. The comparison control group consisted of ten gender- and race-matched controls recruited from the University of Michigan, Ann Arbor, Michigan.

### Clinical and hemodynamic data

Age, race, gender, diagnosis, right heart catheterization pressures, 6-minute hall walk (6MHW) distances, World Health Organization functional capacity classification, platelet counts, and current medication for patients with IPAH were obtained through the PHBI network (Tables 1 and 2).

**Figure 1:**
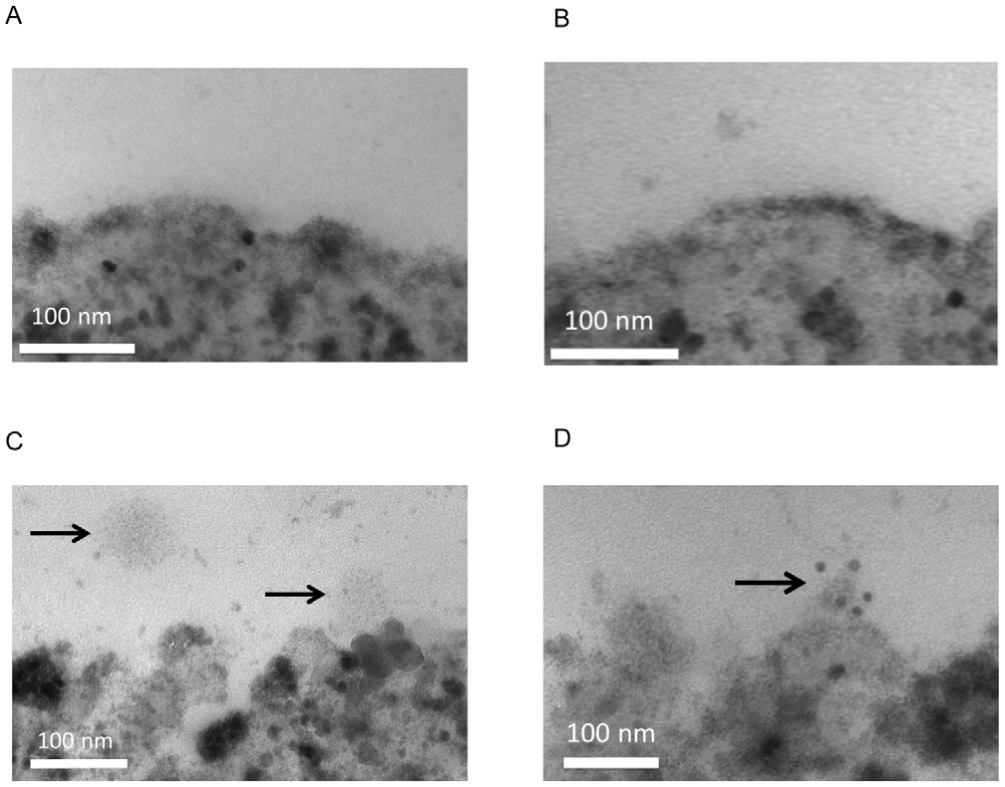
Transmission electron micrographs (TEM) of platelet microparticles (**CD31^+^CD42b^+^**) **with immunogold labeling of surface CD39.** (A) shows the surface of Dynabeads without CD42b capture antibody after exposure to PPP and (B) shows the surface of Dynabeads coated with IgG isotype control after PPP exposure. No microparticles adhered to the surfaces of these Dynabeads. Subsequent lack of immunogold staining for CD39 was also noted. Platelet microparticles were captured by Dynabeads coated with CD42b antibody (arrows on C, D). The sample imaged in C was not incubated with mouse anti-CD39 antibody conjugated to biotin prior to the addition of streptavidin-linked immunogold. (D) shows positive SA-immunogold labeling of a microparticle which was previously incubated with biotinylated anti-CD39. (all at 180,000× magnification).

**Figure 2:**
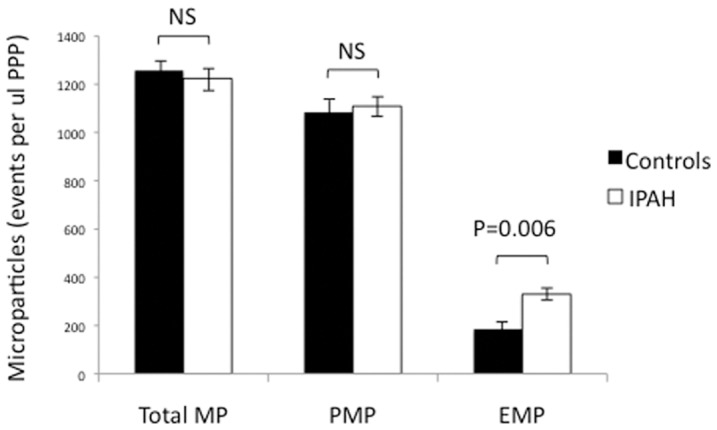
Quantification of circulating microparticles in healthy controls compared to IPAH patients. No difference was found in total microparticle (annexin V^+^) or platelet microparticle (CD31^+^CD42b^+^) levels. Patients with IPAH had a statistically significant increase in circulating endothelial microparticle (CD31^+^CD42b^−^) levels compared to controls. Results expressed as flow cytometric events per ul of platelet poor plasma (PPP) and presented as mean ± SEM. NA  =  not significant, MP  =  microparticles, PMP  =  platelet microparticles, EMP  =  endothelial microparticles.

### Whole blood collection

Antecubital venipuncture using a 21-gauge butterfly needle was used to collect 4.5 mL of whole blood in venous blood collection tubes with 0.5 mL of citrate solution (Becton Dickinson, Franklin Lakes, NJ). In the case of IPAH patients, collection of whole blood occurred at remote sites immediately prior to transplant. The collection tubes were placed in an insulated container containing wet ice, and transferred to our laboratory via overnight courier. To create similar conditions for control samples obtained locally, tubes containing whole blood samples from control patients were placed in insulated containers with wet ice for 16 hours, the average transportation time for IPAH samples.

### Preparation of plasma

Platelet-poor plasma (PPP) was obtained by double centrifugation at room temperature (1,500× g for 25 minutes followed by 15,000× g for 45 minutes). The supernatant containing platelet-poor plasma was transferred to tubes, snap frozen using liquid nitrogen, and stored at −80 degrees Celsius.

**Figure 3:**
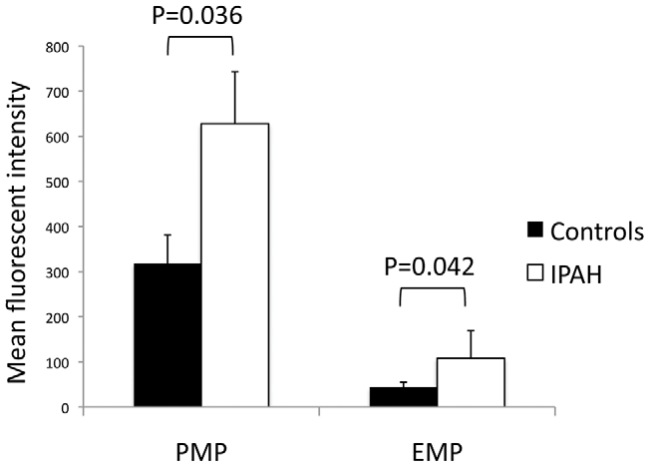
CD39 expression on platelet and endothelial microparticles. Flow cytometry revealed significantly increased surface CD39 expression on platelet (**CD31^+^CD42b^+^**) **and endothelial** (**CD31^+^CD42b^−^**) **microparticles.** PMP  =  platelet microparticles, EMP  =  endothelial microparticles.

### Isolation of plasma microparticles

Frozen samples were thawed at 37 degrees Celsius. Platelet-poor plasma was added to 11×60 mm tubes (Seton Scientific, Los Gatos, California), and ultracentrifuged (ThermoFisher Scientific, Asheville, North Carolina) at 100,000× g for 75 minutes. For each tube, all but 100 µl of the supernatant was removed, and the remaining 100 µl was resuspended in a calcium-free HEPES buffer (10 mM/L HEPES, 5 mM/L KCl, 1 mM/L MgCl_2_, 136 mM/L NaCl) at a pH of 7.4 and used for the experiments described.

**Figure 4:**
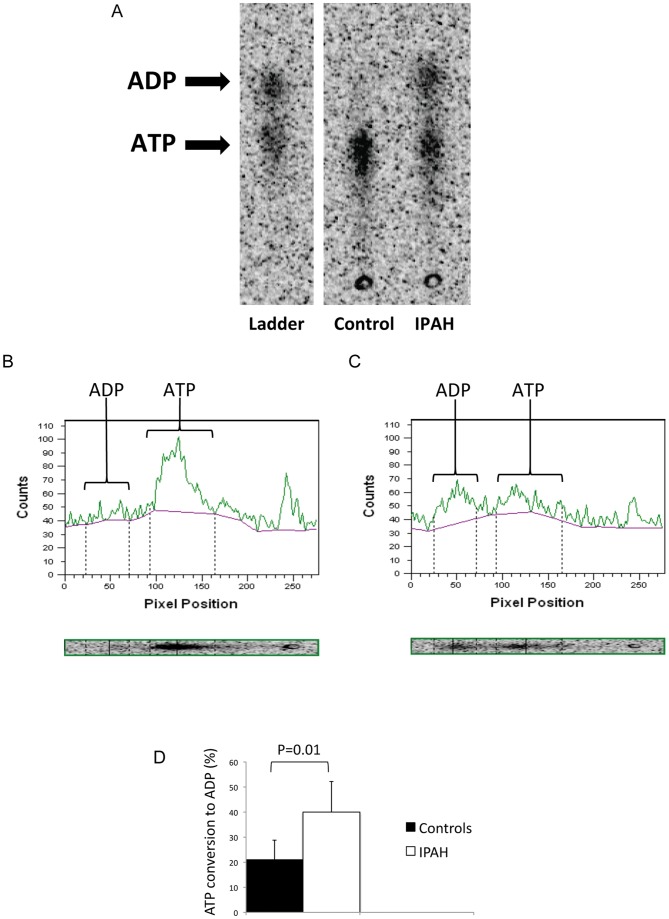
Measurement of CD39 ATPase activity on microparticles from healthy controls compared with IPAH patients. Representative thin layer chromatography (A) shows increased dephosphorylation of 14C-labeled ATP to ADP in samples incubated with microparticles isolated from a patient with IPAH compared to a healthy control. Quantification of radiolabeled ATP and ADP in TLC samples from Controls (B) and IPAH patients (C) revealed a significant increase in the percentage of ATP dephosphorylated to ADP, as shown in (D).

**Figure 5:**
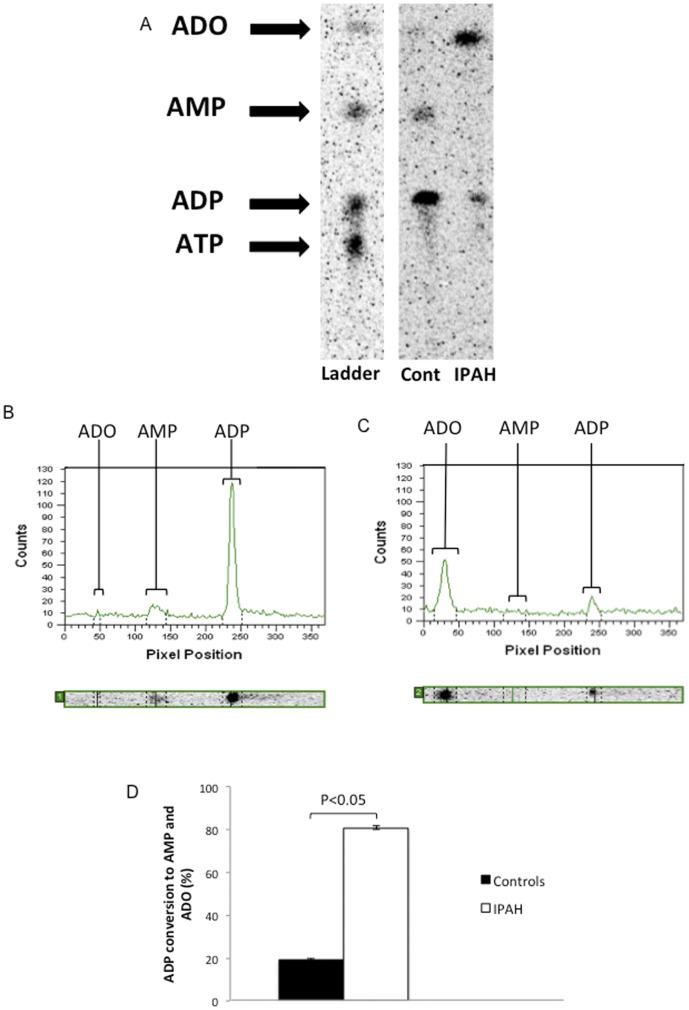
Measurement of CD39 ADPase activity on microparticles from healthy controls compared with IPAH patients. Representative thin layer chromatography (A) shows increased dephosphorylation of 14C-labeled ADP to AMP and adenosine (ADO) in samples incubated with microparticles isolated from a patient with IPAH compared to a healthy control. Quantification of radiolabeled ADP, AMP and ADO in TLC samples from Controls (B) and IPAH patients (C) revealed a significant increase in the percentage of ADP dephosphorylated to AMP and adensoine, as shown in (D).

### Transmission electron microscopy (TEM)

Dynabeads were used to capture platelet microparticles from platelet poor plasma (PPP). The magnetic properties of the Dynabeads ensured a high yield of platelet microparticles despite multiple washing steps. M-270 epoxy Dynabeads were coated with mouse anti-human CD42b antibody to detect the glycoprotein Ib platelet surface antigen. PPP from a human control and coated Dynabeads were added to each of three Eppendorf tubes and mixed. Beads without capture antibody, as well as beads coated with IgG isotype control to correct for Fc binding, were also incubated with PPP as controls. A DynaMag-2 magnet (Invitrogen, Carlsbad, CA) was used to separate the Dynabeads coated with CD42b linked to microparticles from the PPP. The sample was resuspended in phosphate buffered saline (PBS) containing 0.01% TWEEN-20 and biotinylated mouse anti-human CD39 antibody was added. The sample was mixed and the Dynabeads removed using the magnet. The Dynabeads were resuspended in PBS, and Aurion streptavidin-linked 6 nanometer gold particles were added. The sample was mixed on a roller, and the Dynabeads were then separated from the supernatant using the magnet. The sample was resuspended in 0.25% gluteraldehyde phosphate buffer. Each sample was added to an Eppendorf tube containing HistoGel in such a way as to isolate the bulk of the Dynabeads at the tip of the tube. The hardened HistoGel sample was removed and stored in the gluteraldehyde phosphate buffer. Dynabead samples and controls were visualized using a Philips CM-100 electron microscope (FEI, Hillsboro, OR) at magnifications of 10,500 to 180,000X. Processing of samples and TEM imaging was performed by the University of Michigan Microscopy and Image Analysis Laboratory.

**Figure 6:**
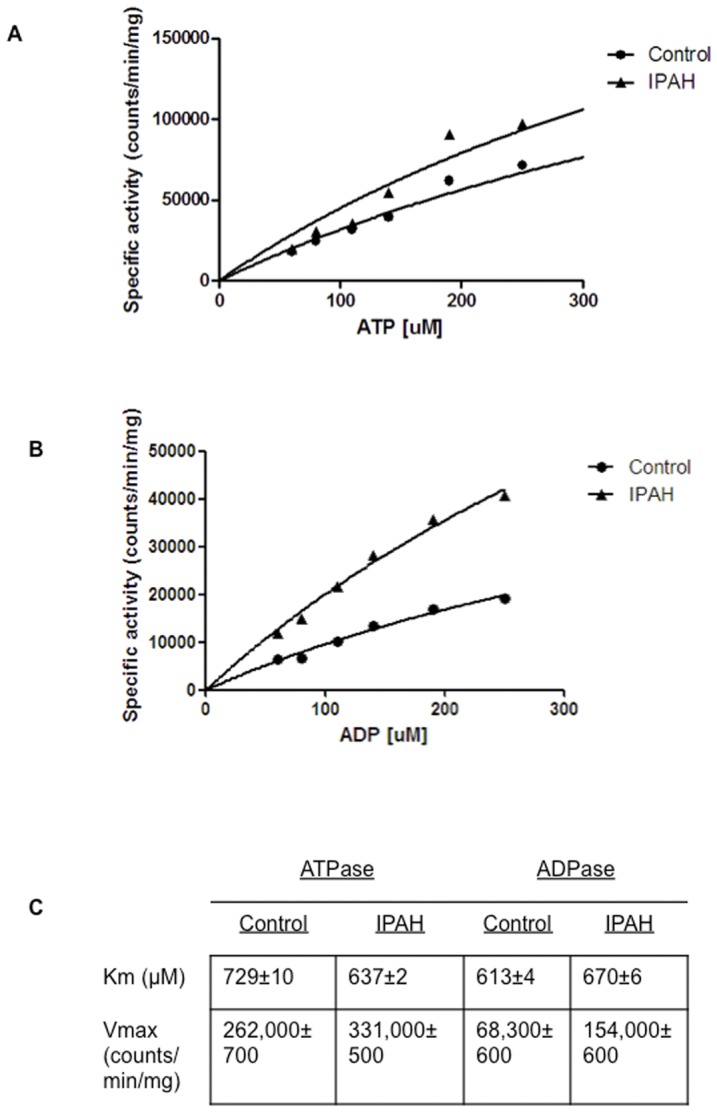
A comparison of ATPase (A) and ADPase (B) activities of microparticles from healthy controls and IPAH patients. Km and Vmax values are shown (**C**). Non-linear regression was used to fit experimental data to the curves using the Mihaelis-Menten equation. Data are means ± SEM from triplicate experiments using microparticles isolated from three healthy controls and three IPAH patients.

### Flow cytometry

A flow cytometer (FACSCalibur, BD Biosciences, San Jose, CA) was used to count the number of microparticle events per milliliter of PPP, and to measure the expression of CD39 on platelet- and endothelial cell-derived microparticles. CaliBRITE beads and FACSComp software (BD, San Jose, CA) were used to check flow cytometer sensitivity and set the voltages and compensation prior to sample evaluation. All samples from patients and controls were processed during the same session using the following protocol. In order to identify the region containing microparticles, FluoSphere fluorescent beads with diameters of 0.5 and 1.5 microns were run using a forward versus side scatter dot plot, and the region between the two bead populations was gated as the population of interest. For total microparticle counts, microparticles isolated from 250 µL of PPP were resuspended in 500 µL of Annexin V Binding Buffer containing calcium. APC-conjugated annexin V was added to the sample and incubated for 30 minutes. The sample was transferred to a TruCOUNT tube containing a lyophilized pellet composed of a known number of fluorescent beads. The previously determined microparticle size gate and a gate around the TruCOUNT beads were used to determine the concentration of annexin-V^+^ microparticles per microliter of PPP using the following equation:

*(number of events in microparticle region/number of events in count bead region) × (number of beads per test/test volume) × a dilution factor of 2 = absolute microparticle count.*

Surface CD39 expression on endothelial and platelet microparticles was identified using triple flurophore-conjugated antibody labeling utilizing FITC-conjugated mouse anti-human CD42b, APC-conjugated mouse anti-human CD31, and PE-conjugated mouse anti-human CD39 antibody added to calcium-free HEPES buffer containing microparticles isolated by ultracentrifugation from 250 µL of PPP. After 30 minutes, 400 µL of HEPES buffer without calcium was added to the sample, and flow cytometry performed. A forward versus side scatter dot plot was used to gate microparticles based on size, as described previously. Triple staining was used to identify CD39 on platelet microparticles (CD39^+^CD31^+^CD42b^+^) and endothelial microparticles (CD39^+^CD31^+^CD42b^−^). The mean fluorescent intensity (MFI) of CD39 was measured on both platelet and endothelial microparticles.

**Figure 7:**
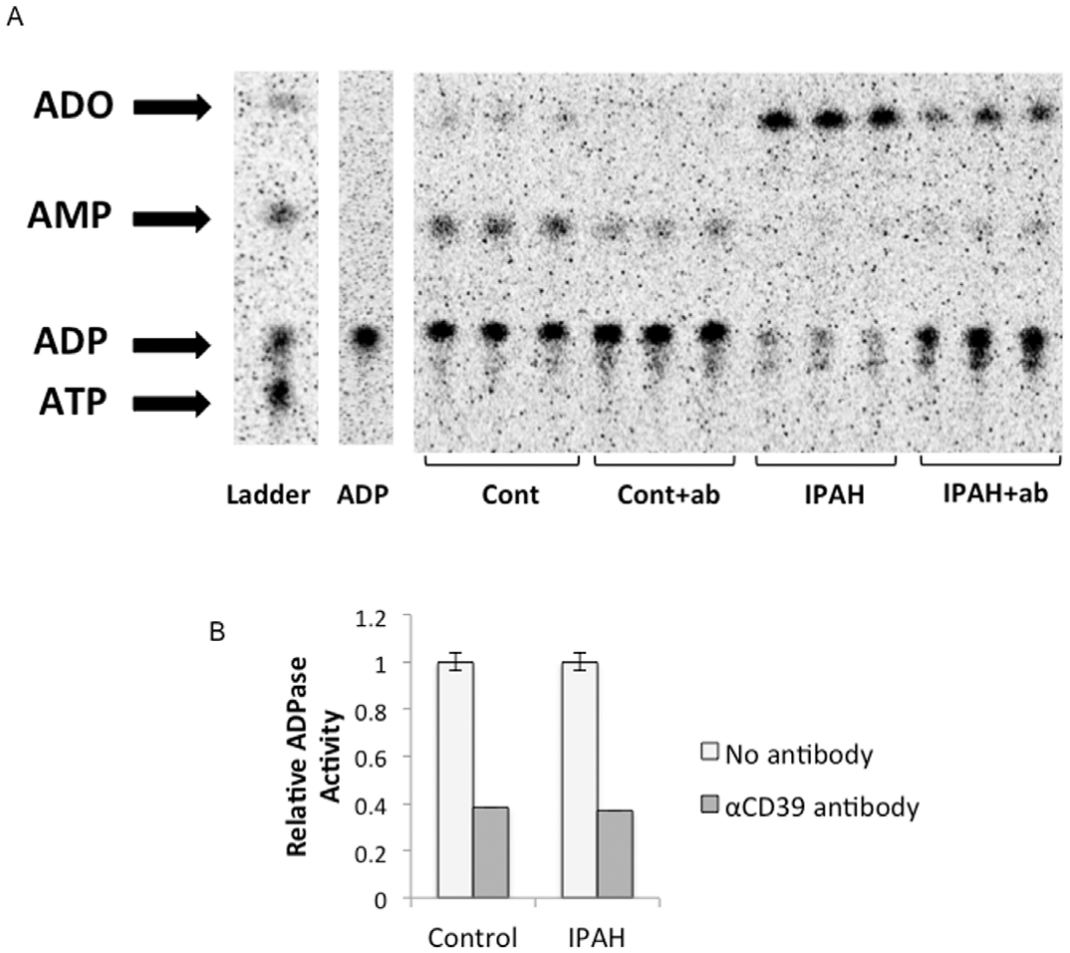
Microparticle ADPase inhibition using anti-CD39 monoclonal antibody. (**A**) TLC performed following anti-CD39 pre-treatment of microparticles from healthy controls and IPAH patients (**Cont+ab** and **IPAH+ab**) shows a decrease in the dephosphorylation of 14C ADP to AMP and adenosine (ADO) in both groups compared to samples without antibody pre-treatment (**Cont** and **IPAH**). The ADP lane contains 14C ADP subjected to the same experimental conditions but without microparticles. Quantification of the percent of available 14C ADP converted to AMP and ADO (**B**) reveals a relative decrease in nucleotidase activity of 62% in controls and 63% in IPAH patients (normalized to samples not pre-treated with antibody).

### Thin layer chromatography

Thin layer chromatography (TLC) was used for enzyme kinetics and inhibitor assays. A previously described liquid phase consisting of isobutanol/isoamyl alcohol/2-ethoxyethanol/ammonia/H_2_O in a 9∶6∶18∶9∶15 ratio [22] was added to a 20cm × 20cm rectangular thin layer chromatography tank (Fisher Scientific, Waltham, MA) and allowed to equalize. For each patient and control sample, microparticles were isolated from 250 µL of PPP using ultracentrifugation (100,000× g at 4 degrees Celsius for 70 minutes), and the concentrated microparticles were resuspended in HEPES buffer. Based on the microparticle count performed for each patient sample (described above), a volume containing 250,000 microparticles was placed in a 1.5 milliliter tube, and the total volume was brought to 25 µL by the addition of PBS, if needed. A substrate mix was created by mixing RPMI containing 2.05 mM L-glutamine with 40–60 mCi/mmol 14C-radiolabeled ATP or ADP. 25 µL of substrate mix was combined with each 25 µL microparticle sample. All tubes were incubated in a water bath at 37 degrees Celsius. Based on time course experiments that identified the linear phase of ATPase and ADPase reactions, 35 minutes was selected as the optimal water bath incubation time for the kinetics and inhibitor assays. A radiolabeled substrate concentration range of 0 to 500 mM was used to determine Km and Vmax for ATPase and ADPase activity. For inhibition assays, 5 µL of each inhibitor (Table 3) was added to the microparticle samples 60 minutes prior to the addition of the radiolabeled substrate. Inhibitors were used at the concentrations shown in Table 3, consistent with other published studies [23], [24]. Each antibody blocking experiment was performed by pre-treating 250,000 isolated microparticles in 5 µL of reaction mixture with 5 µL of one of the following antibodies: anti-CD39 (clone A1, 0.025 mg/mL), anti-ENTPD2 (clone EPR3885, 0.25 mg/mL), anti-ENTPD3 (polyclonal, 0.23 mg/mL), anti-ENTPD8 (polyclonal, 0.25 mg/mL). The remainder of the assay was performed as described above. All steps were performed on ice prior to incubation in the water bath. Kinetic and inhibition assays were performed in triplicate using microparticles isolated from 3 healthy controls and 3 IPAH patients.

Following incubation, 8N formic acid was added to each tube to terminate enzymatic activity. One microliter of each sample, as well as a ladder containing 14C-radiolabeled ATP, ADP, AMP and (in some cases) adenosine were applied to a silica-based TLC plate (Sigma, St Louis, MO) and allowed to dry. The TLC plate was placed in the TLC tank containing the liquid phase, and removed 5 hours later. After drying, the TLC plate was covered in plastic wrap and placed on a storage phosphor screen for two hours. The phosphor screen was then scanned using a phosphorimager (Typhoon, GE Life Sciences, Piscataway, NJ). Quantification of the intensity of TLC spots was performed using ImageQuant software (GE Healthcare, Piscataway, NJ). In order to determine the effects of needle size on nucleotidase activity phlebotomy utilizing antecubital vein access was performed on 3 healthy volunteers using three needle sizes (21G, 23G, 25G). Radiolabeled C-14 ADP was added to one set of reaction mixtures to assess needle size effect on ADPase activity and C-14 ATP was added to another set of reaction mixtures to assess needle size effect on ATPase activity. Km and Vmax were determined using Prism 5.0 software (GraphPad Software, La Jolla, CA) to create a curve based upon the Michaelis-Menten equation.

### Statistical analysis

All data were analyzed using SPSS Statistics (IBM, Somers, NY). Student's t-test was used to compare the mean ± standard error of the mean for variables between groups. Differences were considered significant at *p*<0.05.

## Results

### Demographic and Medical Characteristics of IPAH patient and healthy controls

There were no significant differences in age (*M* = 41 years for controls, *M* = 47 years for IPAH, *p* = 0.7), race, or gender, and no subject was actively using tobacco (Table 1). There was no significant difference in platelet count between the two groups (*M* = 197 for controls, *M* = 183 for IPAH, *p* = 0.72). The medical, functional and hemodynamic characteristics of individuals with IPAH are described in Tables 1 and 2 and are consistent with advanced pulmonary arterial hypertension. Healthy controls were not taking any medications, including aspirin.

### Transmission electron microscopy of microparticles

Circulating platelet microparticles were covalently linked to superparamagnetic Dynabeads. Figure 1A is a 180,000× magnification of a stock Dynabead without capture antibody coating, and Figure 1B is a 180,000× magnification of a Dynabead coated with IgG isotype control. Note the lack of microparticles bound to the surfaces, indicating that non-specific binding of platelet microparticles does not occur. These samples were also incubated with immunogold particles linked to streptavidin (SA). The lack of gold particles shows that nonspecific binding of SA to the bead surface does not occur. Figure 1C is a 180,000x magnification of a Dynabead coated with mouse anti-human CD42b antibody against platelet GP1b platelet surface antigen and then incubated with microparticles. The arrows identify a microparticle in the vicinity of the Dynabead (left) as well as a platelet microparticle captured on the surface (right). The sample was also exposed to SA-linked immunogold particles without having first been incubated with biotinylated mouse anti-human CD39. Note the lack of immunogold labeling of the microparticles in this figure, indicating that nonspecific binding of SA to microparticles does not occur. Figure 1D illustrates the result of pretreating platelet microparticles bound to Dynabeads with biotinylated mouse anti-human CD39 prior to the addition of SA-linked immunogold particles. The arrow identifies a bound microparticle with 6nm gold particles linked to surface CD39 through streptavidin-biotin interaction.

### Quantification of circulating microparticles by flow cytometry

Measurements of circulating total microparticles, as well as platelet and endothelial microparticle subtypes, are shown in Figure 2. The results are given in units of flow cytometric “events per microliter of PPP.” In the case of the first pairing of bars on the left side of Figure 2, total microparticles were identified using annexin-V conjugated to the allophycocyanin fluorophore. Note that there is no statistically significant difference in the number of total microparticles circulating in patients with IPAH versus controls (1224±205 events/µl PPP vs. 1257±89 events/µl PPP). The number of circulating platelet microparticles (CD31^+^CD42b^+^) in IPAH patients and controls was also similar (1109±174 events/µl PPP vs. 1085±73 events/µl PPP). A statistically significant increase in circulating endothelial microparticles (CD31^+^CD42b^−^) was identified in patients with IPAH compared to healthy controls (330±32 events/µl PPP vs. 185±27 events/µl PPP), as shown by the pairing of bars to the right in Figure 2.

### Flow cytometric comparison of microparticle surface CD39 expression

Figure 3 shows the mean fluorescent intensity of CD39 on circulating platelet microparticles (CD39^+^CD31^+^CD42b^−^) and endothelial microparticles (CD39^+^CD31^+^CD42b^+^), as determined by flow cytometry. As shown in the pairing of bars on the left, platelet microparticles from patients with IPAH have a statistically significant two-fold increase in CD39 expression compared to controls. The bars on the right of Figure 3 reflect the significant 2.5-fold increase in CD39 expression found on circulating endothelial microparticles from patients with IPAH compared to healthy controls.

### Measurement of microparticle nucleotidase kinetics and inhibition by thin layer chromatography

Thin layer chromatography was used to separate carbon-14-labeled nucleotides after varying degrees of dephosphorylation by microparticle nucleotidase activity. Subsequent scanning of the TLC plates with a phosphorimager allowed for the quantification of ATP, ADP, AMP, and adenosine in each sample. As shown in Figure S1, differences in needle gauge did not produce a significant difference in the percentage of radiolabeled ATP or ADP dephosphorylayted. Figure 4 shows a representative plate assessing the ATPase activity of microparticles from one healthy control and one IPAH patient. No AMP or ADO signals were detected. Each of the processed plates containing samples from the remaining healthy controls and IPAH patients had similar results. The narrow left panel shows the ladder containing known radiolabeled nucleotides to facilitate identification of each nucleotide in the patient samples. The ladder mixture contains 14C-labeled nucleotides and adenosine, and is added to the plate at the same time as the patient samples. In Figure 4A, ADP has migrated more rapidly than ATP, and is thus closer to the top of the plate. The wider dual-lane panel on the right of Figure 4A shows the migration of samples from healthy controls and patients with IPAH. The left lane contains the nucleotide mixture that had been incubated with 250,000 microparticles from a healthy control. The lane containing the reaction mixture incubated with microparticles from a patient with IPAH shows spots with similar intensity in both the ATP and ADP regions. Thus, more ATP was dephosphorylated to ADP in the IPAH sample compared to the control sample, indicating increased ATPase activity. Figure 4B illustrates a quantitative scan of the data shown in 4A. The line running continuously underneath the contours of the histogram represents the background signal of the TLC plates, and is subtracted from the final volumes calculated for each nucleotide spot. The narrow, horizontal rectangle beneath the histogram is a smaller version of the TLC lane from Figure 4A, and aids in identifying the region of the spot to be quantified. Figure 4C shows the quantification of the ADP and ATP peaks for the IPAH lane shown in Figure 4A. Figure 4D shows the mean percentage of ATP dephosphorylated to ADP over the course of 35 minutes by microparticles for the ten healthy controls and ten IPAH patients studied. There is a statistically significant (50%) increase in microparticle ATPase activity in IPAH patients compared to controls (43.8±11.0% vs. 29.9±7.6%, p = 0.01). Figure 5 compares ADPase activity of a healthy control versus that of an IPAH patient following the addition of 14C ADP to a reaction mixture containing 250,000 microparticles. As shown, microparticles from IPAH patients dephosphorylated 400% more 14C ADP to AMP and adenosine than the control (80.8±0.8% vs. 19.0±0.83%, p<0.05).

The results of ATPase and ADPase kinetic assays are shown in Figure 6. At the chosen incubation time of 35 minutes about 28% of ATP and 25% of ADP are dephosphorylated, indicating that near-initial rate conditions are satisfied. Figures 6A and 6B show the data fit to Michaelis-Menten curves. The apparent K_m_ for the dephosphorylation of ATP by microparticles from controls and IPAH patients was of essentially the same magnitude (729±10 μM and 637±2 μM, respectively). Similarly, the apparent K_m_ for the dephosphorylation of ADP by microparticles from controls and IPAH patients was 613±4 μM and 670±6 μM, respectively. The V_max_ for the dephosphorylation of ATP by microparticles from IPAH patients was significantly greater than the Vmax for controls (331,000±500 versus 262,000±700 counts per minute per mg of microprticles, respectively). Similarly, the V_max_ for the dephosphorylation of ADP by microparticles from IPAH patients and controls was quite different (154,000±600 versus 68,300±600 counts per minute per mg of microprticles, respectively). A comparison of the kinetics data in Figure 6C shows that the difference in ATP and ADP nucleotidase activity in controls versus patients with IPAH result in a substantial increase in V_max_ with minimal change in K_m_.

To determine whether the predominant microparticle nucleotidase is consistent with an ectonucleoside triphosphate diphosphohydrolase (ENTPD), specifically CD39 (ENTPD-1), inhibitor studies were performed. Ouabain [25] was used to inhibit Na^+^/K^+^ ATPase activity, and the mitochondrial ATPase was inhibited with sodium azide and oligomycin [26]. The acid phosphatase inhibitor ammonium molybdate (also an inhibitor of 5′-nuceotidases), the Ser/Thr phosphatase inhibitor beta-glycerophosphate, and the alkaline phosphatase inhibitor levamizole were also tested. As shown in Table 3, there was no effect of any of these inhibitors on either ATPase or ADPase activity. However, the addition of the non-penetrating ectonucleotidase inhibitor suramin [27], the selective ecto-ATPase inhibitor ARL 67156 [28] or POM-1, a selective inhibitor of ENTPD-1, -2, and -3 [29], significantly decreased ATPase and ADPase activity on both control and IPAH microparticles.

To further identify CD39 as the predominant microparticle ectonucleotidase, antibody blocking experiments were performed using antibodies against ENTPD-1 (CD39), ENTPD-2, ENTPD-3, and ENTPD-8. The chromatogram in Figure 7A shows ADPase activity after 60 minutes of pre-treatment of microparticles with the anti-CD39 antibody. Microparticles from both healthy controls (**Cont**) and IPAH patients (**IPAH**) dephosphorylated less 14C ADP to AMP and adenosine (**ADO**) after pre-treatment with the anti-CD39 antibody. The relative ADPase activity of microparticles isolated from control and IPAH patients decreased by 62% and 63%, respectively, after pre-treatment with αCD39 antibody (Figure 7B). In order to rule out a significant role for related ENTPDases, experiments were performed using pre-treatment with antibodies against ENTPD-2, -3, and -8. These antibodies produced no change in ADPase or ATPase activity (data not shown).

## Discussion

The current study characterizes, for the first time, the nucleotidase activity of microparticles isolated from healthy controls and patients with IPAH. In addition, we demonstrate that microparticles isolated from IPAH patients have increased ATPase and ADPase activity, as well as increased surface CD39 expression. Our finding of similar ATPase and ADPase apparent Km values for control and IPAH microparticles suggests that it is the same enzyme present on the surface of microparticles of both groups. Additionally, the ratio of ATPase to ADPase Vmax values of 3.9 (control) and 2.1 (IPAH) is similar to the 2.5-fold difference found by Zebisch et al in their characterization of CD39 refolded from bacterial inclusion bodies [30]. This is consistent with CD39 being the primary ATP and ADP nucleotidase on the microparticles. The role of an ENTPDase is more directly supported by inhibitor blocking studies. Antibody blocking experiments further identify CD39 as a significant microparticle ENTPDase. TEM showing positive immunogold labeling of CD39 on the surface of circulating platelet microparticles also supports the presence of CD39 on the surface of microparticles. We chose to capture the platelet-derived subtype of microparticle for TEM because it comprises the majority of circulating microparticles [18].

The finding of a significant increase in microparticle CD39 may have clinical implications. Earlier work by Banz et al [16] and Bakouboula et al [31] support the hypothesis that microparticle compositions have systemic effects, and that alterations in microparticle phenotype may be related to pathological states. In the case of IPAH, an increase in circulating microparticle CD39 activity may result in a shift in the intravascular nucleotide/nucleoside milieu away from an ATP- and ADP-laden environment. ATP has been shown to have a potent endothelium-dependent vasodilatory effect on human pulmonary arteries *via* its ability to stimulate nitric oxide [32] and prostacyclin [33] release from vascular endothelium. Treatment with infusions of ATP-MgCl_2_ has been shown to be a safe and effective treatment for pulmonary hypertension associated with congenital heart defects in children [6]. Decreased levels of both prostacyclin and nitric oxide have been found in patients with IPAH, and exogenous administration of them are validated therapies for patients with the disease.[2] Thus, a decrease in intravascular ATP concentration through increased microparticle ATPase activity may contribute to the increased pulmonary vascular resistance measured in patients with IPAH.

The increased microparticle surface CD39 described in our study may instead reflect a compensatory mechanism activated by the body in an attempt to mitigate the pro-inflammatory, pro-thrombotic, hyper-proliferative processes associated with IPAH. An increase in ATP and ADPase activity shifts the intravascular nucleotide composition towards increased AMP. AMP is the substrate for ecto-5′ nucleotidase (CD73), a membrane bound glycoprotein that dephosphorylates AMP into adenosine [34]. Adenosine is a known vasodilator [35] that also modulates platelet aggregation [36], inflammation [37], and cell proliferation [38]. Platelet-derived ADP is a potent stimulator of platelet aggregation [9] and thrombus formation [39], and acts in a synergistic fashion with peptide growth factors to induce SMC proliferation [40]. Thus, CD39- and CD73-mediated conversion of ATP and ADP into AMP and ultimately adenosine may at once deprive the intravascular milieu of pro-thrombotic, pro-inflammatory nucleotides and increase the availability of a vasodilatory, anti-inflammatory, anti-thrombotic, and anti-proliferative nucleoside.

Our studies show an increase in the number of circulating endothelial microparticles, but no difference in total or platelet microparticles in patients with IPAH compared to healthy controls. These findings are consistent with those of Amabile et al, who identified circulating endothelial microparticles as CD31^+^CD41^−^ (glycoprotein IIB negative) by flow cytometry [41]. Our measured total, endothelial, and platelet microparticle concentrations are lower than those in this earlier study, and may be secondary to differences in flow cytometric technique or a lesser degree of systemic activation.

Our study has several limitations. Our sample size of ten patients with IPAH reflects the rarity of this disease. The creation of the PHBI network has enabled researchers to share invaluable tissue from patients, and continued collaboration through the network will allow us to increase the sample size of future experiments. Our IPAH samples were shipped to our laboratory, arriving one day after phlebotomy. While this did not affect microparticle concentration or nucleotidase activity, intravascular nucleotides are known to degrade rapidly. Thus, the actual concentrations of nucleotides in the blood samples were not measured in these studies. Future investigations will measure intravascular nucleotide concentrations in IPAH patients recruited from our institution, allowing for rapid measurement soon after phlebotomy.

In conclusion, our investigation utilizes kinetic, inhibition, and TEM studies to identify CD39 as an important nucleotidase on circulating microparticles. We also demonstrate increased CD39 expression on the surface of circulating platelet (CD31^+^CD42b^+^) and endothelial (CD31^+^CD42b^−^) microparticle subpopulations in patients with IPAH. We have shown increased microparticle ATPase and ADPase activity in patients with this disease, indicating that the increased surface CD39 is functional. Further studies are needed to determine whether the increase in functional microparticle-bound CD39 contributes to the pathogenesis of IPAH, or whether it represents a compensatory mechanism aimed at mitigating the devastating effects of this rapidly fatal disease.

## Supporting Information

Figure S1 **Needle gauge used for phlebotomy does not alter CD39 activity on microparticles.** Phlebotomy utilizing antecubital vein access was performed on 3 healthy volunteers using three needle sizes (21G, 23G, 25G). Radiolabeled 14C ADP was added to one set of reaction mixtures to assess needle size effect on CD39 **ADPase** activity and 14C ATP was added to another set of reaction mixtures to assess needle size effect on CD39 **ATPase** activity (**A**). Differences in needle gauge did not produce a significant difference in the percentage of radiolabeled ADP (**B**) or ATP (**C**) dephosphorylayted. Isolation of microparticles, TLC, and quantification of enzyme activity were performed as described in the Methods section of primary manuscript.
(TIF)
