# Supplementary figures and images for: Increased CD39 Nucleotidase Activity on Microparticles from Patients with Idiopathic Pulmonary Arterial Hypertension
Source: PLoS One. 2012 Jul 11;7(7):e40829. doi: 10.1371/journal.pone.0040829 (PMC3394716; doi:10.1371/journal.pone.0040829)

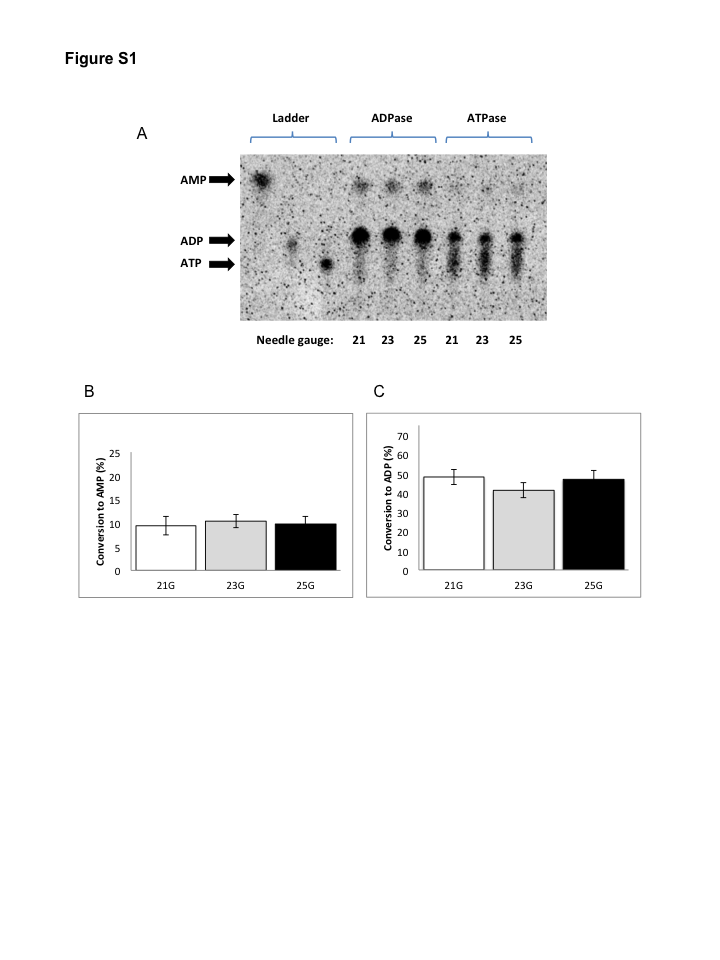

Supplement: Figure S1 — Needle gauge used for phlebotomy does not alter CD39 activity on microparticles. Phlebotomy utilizing antecubital vein access was performed on 3 healthy volunteers using three needle sizes (21G, 23G, 25G). Radiolabeled 14C ADP was added to one set of reaction mixtures to assess needle size effect on CD39 ADPase activity and 14C ATP was added to another set of reaction mixtures to assess needle size effect on CD39 ATPase activity (A). Differences in needle gauge did not produce a significant difference in the percentage of radiolabeled ADP (B) or ATP (C) dephosphorylayted. Isolation of microparticles, TLC, and quantification of enzyme activity were performed as described in the Methods section of primary manuscript. (TIF) [file pone.0040829.s001.tif]
